# Assessing the Efficacy of Lopinavir/Ritonavir-Based Preferred and Alternative Second-Line Regimens in HIV-Infected Patients: A Meta-Analysis of Key Evidence to Support WHO Recommendations

**DOI:** 10.3389/fphar.2018.00890

**Published:** 2018-08-14

**Authors:** Yinqiu Huang, Xiaojie Huang, Yadong Luo, Yihong Zhou, Xingbao Tao, Hui Chen, Aixin Song, Yaokai Chen, Hao Wu

**Affiliations:** ^1^National Key Laboratory for Infectious Diseases Prevention and Treatment With Traditional Chinese Medicine, Chongqing Public Health Medical Center, Chongqing, China; ^2^Center for Infectious Diseases, Beijing You'an Hospital, Capital Medical University, Beijing, China; ^3^Center for Infectious Diseases, Chongqing Public Health Medical Center, Chongqing, China; ^4^School of Biomedical Engineering, Capital Medical University, Beijing, China

**Keywords:** lopinavir/ritonavir, human immunodeficiency virus, second-line antiretroviral therapy, ART-experienced, efficacy and safety

## Abstract

**Background:** Nucleoside reverse transcriptase inhibitors (NRTIs) and non-NRTIs (NNRTIs) with boosted protease inhibitors are included in standardized first-line and second-line regimens. Recent World Health Organization (WHO) guidelines recommend a boosted protease inhibitor (PI) combined with 2 NRTIs or raltegravir as a second-line regimen.

**Objective:** Ritonavir-boosted lopinavir (LPV/r) is known as a key second-line antiretroviral therapy (ART) in resource-limited settings. We carried out a meta-analysis to analyze virologic suppression and effectiveness of LPV/r-based second-line therapy in HIV-infected patients.

**Methods:** In this meta-analysis, we searched randomized controlled trials and observational cohort studies to evaluate outcomes of second-line ART for patients with HIV who failed first-line therapy. A systematic search was conducted in Pubmed, Cochrane Library, and Embase from inception to January 2018. Outcomes included viral suppression, CD4 cell counts, drug resistance, adverse events, and self-reported adherence. We assessed comparative efficacy and safety in a meta-analysis. Data analysis was performed using RevMan 5.3 and Stata12.0.

**Results:** Nine studies comprising 3,923 patients were included in the meta-analysis. The overall successful virologic suppression rate of the second-line regimen was 77% (ITT) and 87% (PP) at 48 weeks with a plasma HIV RNA load of <400 copies/mL. No statistical significance was found in CD4 cell count recoveries between LPV/r plus 2-3 NRTIs and simplified regimens (LPV/r plus raltegravir) at 48 weeks (*P* = 0.09), 96 weeks (*P* = 0.05), and 144 weeks (*P* = 0.73). Four studies indicated that the virus had low-level resistance to LPV/r, and the most common clinically significant PI-resistance mutations were 46I, 54V, 82A/82F, and 76V; however, no virologic failure due to LPV/r resistance was detected. In addition, no statistical significance was found between the two groups in self-reported adherence [relative risks (RR) = 1.03,95% confidence interval (CI) 1.00, 1.07, *P* = 0.06], grade 3 or 4 adverse events (RR = 0.84, 95% CI 0.64, 1.10, *P* = 0.20) or serious events (RR = 0.85, 95% CI 0.77, 1.17, *P* = 0.62).

**Conclusions:** These results suggest that the LPV/r-based regimen demonstrates efficacious and low resistance as second-line antiretroviral therapy.Both LPV/r plus 2-3 NRTIs and LPV/r plus RAL regimens improved CD4 cell counts. There was no evidence of superiority of simplified regimens over LPV/r plus 2-3 NRTIs.

## Introduction

Millions of adults and children who are infected with HIV in resource-limited regions have access to antiretroviral therapy (ART) based on World Health Organization (WHO) guidelines (Gilks et al., 2006; World Health Organization, 2013). With the development of antiretroviral drugs, HIV infection has changed from a fatal disease to a chronic disease (Palella et al., 1998; Schackman et al., 2006). The promotion and use of first-line ART in resource-limited settings has significantly reduced the mortality of patients and prolonged life expectancy (Mills et al., 2011) for people living with HIV/AIDS (PLWHA). However, in recent years, a growing number of patients have presented with first-line regimen (World Health Organization, 2013) failure and have been switched to second-line therapy (Long et al., 2010; Hamers et al., 2011; Hoen et al., 2011). Assessing the optimum second-line ART regimen in PLWHA who experience first-line therapy failure in resource-limited settings has important clinical, public health, and health policy considerations. WHO recommends a ritonavir-boosted protease inhibitor (PI) (either lopinavir or atazanavir)-based regimen, and this regimen has been used for preferred standardized (LPV/r plus 2-3 NRTI) or simplified alternative (LPV/r plus RAL) second-line therapy (2016). LPV/r is widely used in resource-limited regions, such as China. LPV/r has been previously shown to have antiretroviral activity and durable tolerability, and it has been approved for use in ART-naïve and experienced patients in combination with other ART drugs. However, there is limited knowledge of the treatment outcomes of LPV/r-based second-line ART in resource-limited settings (Harbord et al., 2006; Humphreys et al., 2007; Gathe et al., 2009).

Growing evidence indicates that LPV/r can be used successfully in ART-experienced patients. Several studies showed that PI-based second-line treatment can effectively reduce viral loads and increase CD4 cell counts and is a cost-effective approach to prevent the spread of drug-resistant HIV. Currently, boosted PI options in second-line regimens are recommended with respect to safety and efficacy in some systematic reviews and network meta-analyses, but there is no solid evidence for the safety and efficacy of LPV/r in second-line therapy (Kanters et al., 2017). Therefore, we conducted this systematic review and meta-analysis of the effectiveness of second-line ART with LPV/r (preferred or alternative regimen) for all patients who experienced first-line ART failure.

## Methods

This article was reported according to the Preferred Reporting Items for Systematic Reviews and Meta-Analyses (PRISMA) statement (Moher et al., 2010), registration number CRD42017074651 (http://www.crd.york.ac.uk/PROSPERO/display_record.php?ID=CRD42017074651).

### Data sources and selection criteria

Systematic searches were conducted in Cochrane Library, Pubmed, and Embase databases for publications in English with key search words, including “HIV” and “ritonavir-boosted lopinavir.” Reference lists of review articles identified in our searches were also checked. This search period ranged from inception to January 2018. One author (Y.Q. HUANG) screened all abstracts. Two authors (Y.Q. HUANG and Y.D. LUO) obtained full-text copies of relevant publications that reported viral suppression, CD4 cell counts and other outcome results, assessed those articles for eligibility and reached a consensus on potential relevance. We included studies of HIV treatment experienced patients over 12 years old who failed first-line NRTI-based regimens and were switched to LPV/r plus 2-3 NRTI regimens. According to the following criteria, certain studies were excluded: (1) reviews and animal studies, (2) regimens without LPV/r, (3) patients living with HIV who were less than 12 years old or pregnant, (4) patients with opportunistic infections and not treated with second-line therapy, and (5) reports without baseline CD4 cell counts or viral load monitoring.

### Data extraction

According to the consensus list, one investigator extracted the data. The data included second-line ART regimens, treatment failure definition and follow-up durations of second-line treatment, viral load monitoring, baseline CD4 cell counts, adherence and drug resistance. When there was uncertainty about the data, the original study authors were contacted for clarification. Detailed data, such as study design, participant information, interventions, outcomes, and adverse events, were extracted and imported into an Excel table. A second author randomly checked 25% of all the extracted data (Kelly et al., 2018). Any discrepancies between investigators were resolved through discussion until consensus was reached.

### Study quality assessment

We used the Cochrane risk of bias tool (Higgins et al., 2011) to evaluate the methodological quality of RCTs included in this meta-analyses. Random sequence generation and allocation concealment representing selection bias, participants and personnel blinding representing performance bias, and outcome data blinding were included in our assessment. Studies meeting all of the criteria were considered to have a low risk of bias, whereas those that met none of the criteria were considered to have a high risk of bias. Other studies were classified as having an unclear risk of bias if the information was insufficient to make a judgment.

### Statistical analysis

We used published estimated relative risks (RRs) provided in study reports and calculated the RR for dichotomous outcomes and the 95% confidence interval (CI) when necessary. We then pooled data across the included studies and estimated their summary effect sizes. We performed all meta-analyses with RevMan 5.3 (The Nordic Cochrane Center, The Cochrane Collaboration, Copenhagen, 2014) and Stata12.0 (StataCorp LP, USA). Heterogeneity for each combined rate was assessed with a chi-square-based *Q*-test and the *I*^2^ statistic. Heterogeneity was considered moderate to large when *P* < 0.1 for a *Q*-test or *I*^2^> 50%. In our meta-analyses, we used random effects sizes models when heterogeneity was moderate to large and fixed effects models when heterogeneity was small.

## Results

### General study information

**Figure 1:**
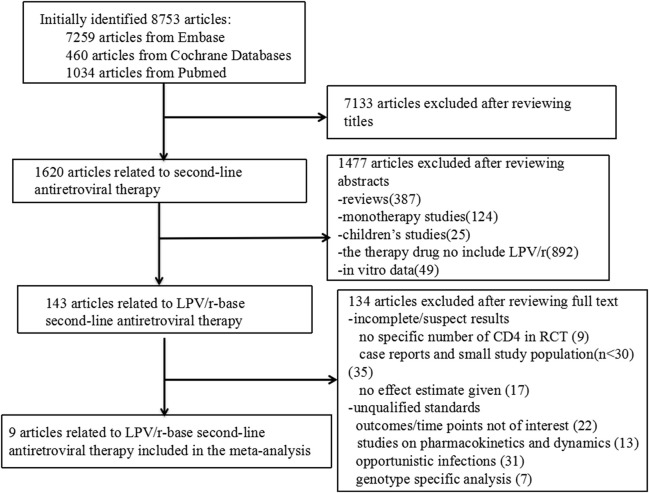
Flow diagram of study selection.

**Figure 2:**
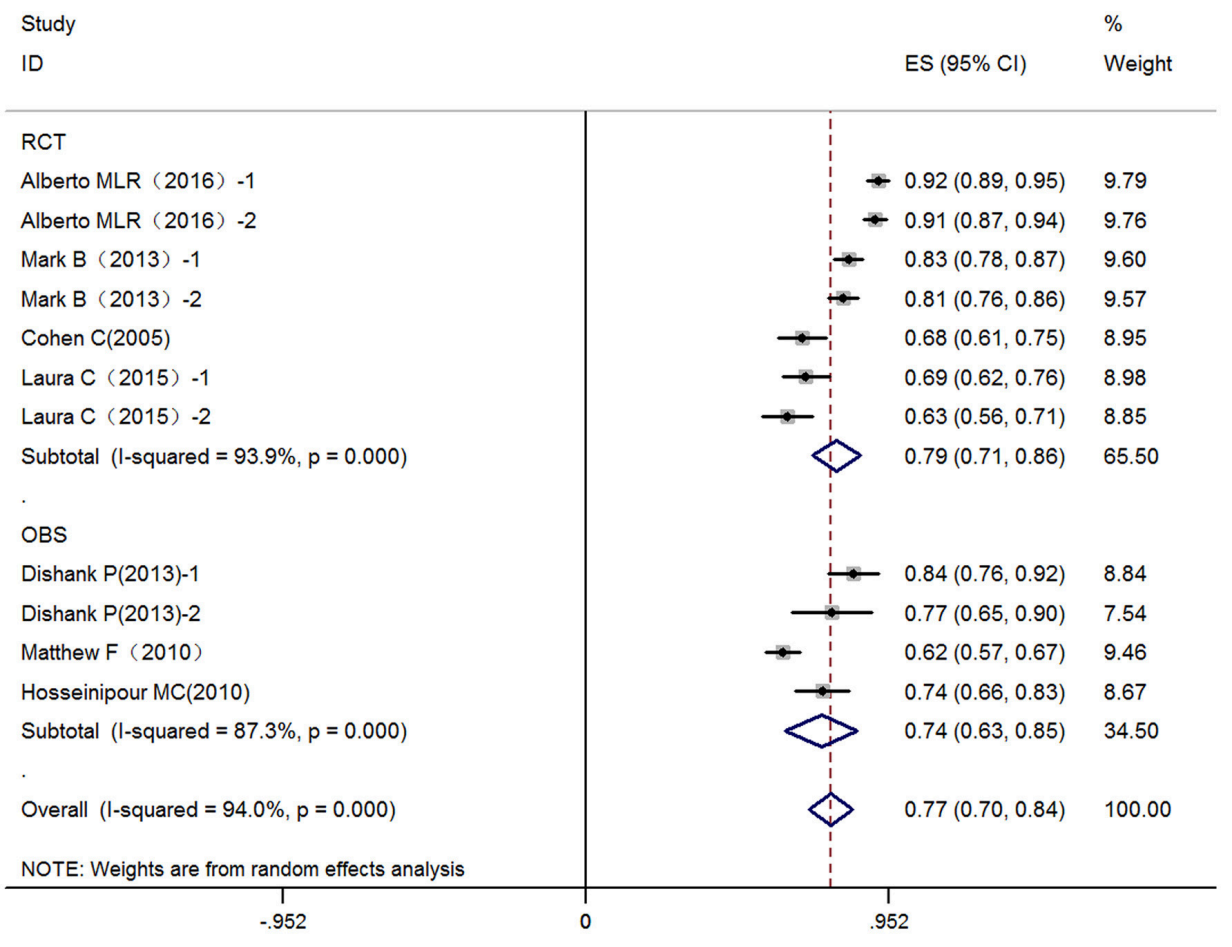
The different between RCT and observational study in virologic suppression. Data are the difference in virologic suppression for RCT and observational research. 1, treatment group: LPV/r plus RAL (NRTI); 2, control group: LPV/r plus NRTis; OBS, observational study; CI, credible interval; NRTI, nucleoside/nucleotide reverse transcriptase inhibitor; RAL, Raltegravir; LPV/r, Ritonavir-boosted lopinavir.

According to our search strategy, we identified 8,753 articles, specifically, 7,259 articles from Embase and 1,494 articles from Cochrane and Pubmed databases. After reviewing the titles and abstracts, 8,610 studies were excluded. The remaining 143 studies assessed LPV/r-based second-line antiretroviral therapy to treat HIV-infected patients who failed first-line antiretroviral therapy. Finally, 134 studies were excluded from our meta-analysis after detailed review for various reasons. Therefore, 9 (Cohen et al., 2005; Fox et al., 2010; Hosseinipour et al., 2010; Patel et al., 2013; SECOND-LINE Study Group et al., 2013; Paton et al., 2014; Ciaffi et al., 2015; La Rosa et al., 2016; Hakim et al., 2018) articles involving 3,773 patients who failed first-line antiretroviral therapy and switched to LPV/r-based second-line ART were used in the present meta-analyses. The literature search strategy and information for these studies are shown in Figure 1 and Table 1, respectively. Three studies mentioned ethnic origin. There was no significant difference between the effect of LPV/r combined with RAL or 2-3 NRTIs and LVP/r plus 2-3 NRTIs and ethnic origin (RR 1.00, 95% CI 0.92, 1.09, *P* = 1.00) (Figure 2).

**Table 1:** General information for the studies included in the meta-analysis.

**Authors & year**	**Antiretroviral therapy (Experimental group/Control group)**	**Number of participants**	**Study duration (weeks)**	**Baseline viral load (range) (log)**	**Baseline CD4 cell count (cell/μL)**	**ΔCD4 count (cell/μL±SD)**	**Study design**	**Ethnic origin (Experimental group/Control group)**
Ciaffi et al., 2015	LPV/r+TDF+FTC LPV/r+ABC+ddi	145 152	48	4.4 (4.0–5.0) 4.6 (4.1–5.1)	199 195	127^*^	RCT	Bobo Dioulasso (30/28) Dakar (21/18) Yaounde (101/99)
SECOND-LINE Study Group et al., 2013	LPV/r+2–3NRTIs LPV/r+RAL	271 270	48	4.3 (3.7–4.9) 4.2 (3.6–4.8)	189 190	132.5 ± 146.0 167.4 ± 142.1	RCT	White (18/23) Asian (117/112) Hispanic (38/37) African (98/97) Unknown (0/1)
Paton et al., 2014	LPV/r+2-3NRTIs LPV/r+RAL	426 433	96	4.8 (4.4–5.2) 4.9 (4.4–5.3)	72 70	234.0 ± 208.1 260.0 ± 185.5	RCT	NA
La Rosa et al., 2016	LPV/r+2-3NRTIs LPV/r+RAL	254 258	48	4.6 (3.6–5.4) 4.5 (3.6–5.4)	178 182	190.0 ± 133.6 199.0 ± 131.2	RCT	White (1/0) Black (162/164) Hispanic (9/11) Asian or Pacific Islander (82/83)
Hosseinipour et al., 2010	LPV/r+ZDV+3TC +TDF	101	48	NA	66	142.0 ± 32.8	Observational	NA
Fox et al., 2010	LPV/r+AZT+ddi	328	48	NA	84	133.0 ± 158.9	Observational	Black (309) Other (19)
Patel et al., 2013	LPV/r+ZDV+3TC +TDF LPV/r+3TC+TDFr	82 44	48	5.4 (5.2–5.5) 5.3 (5.2–5.4)	137 99	204 226	Observational	NA
Cohen et al., 2005	LPV/r+2 NRTIs ATV/r+2 NRTI	150 150	48	NA NA	256 288	169 112	RCT	Hispanic/Latino(52/51) White(41/43) Black(7/6) Asian/Pacific Islander(0/1)
Hakim et al., 2018	LPV/r+2-3NRTIs LPV/r+RAL	426 433	144	4.8 (4.4–5.2) 4.9 (4.4–5.3)	72 70	290.0 ± 247.6 296.0 ± 258.9	RCT	NA

*RCT, randomized controlled trial; SD, standard deviation; NRTI, nucleoside/nucleotide reverse transcriptase inhibitor; RAL, raltegravir; LPV/r, ritonavir-boosted lopinavir; AZT, azidothymidine; TDF, tenofovir disoproxil fumarate; FTC, emtricitabine; ABC, abacavir; ddi, didanosine; ZDV, zidovudine; 3TC, lamivudine; ATV, ritonavir-boosted atazanavir*.

* *ΔCD4 cell counts in two groups*.

### Assessment of the bias risk of the inclusion studies

**Figure 3:**
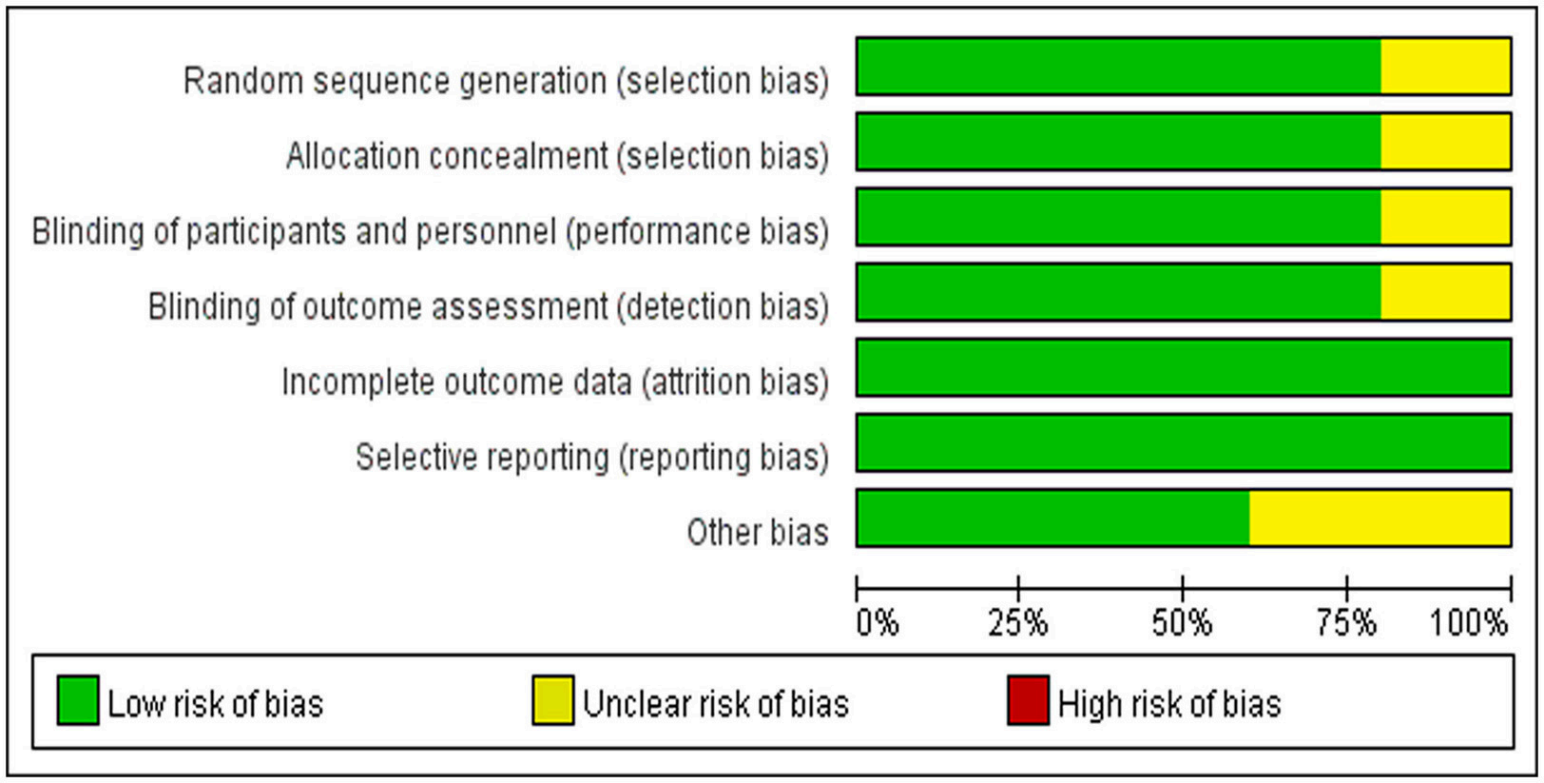
Risk of bias in included RCTs.

HUANG and LUO independently assessed the methodological quality of the included RCTs by applying the bias tool of Cochrane Collaboration's risk. Reviewers rated the quality of all the treatment effects by using the four-step approach proposed in Grading of Recommendations Assessment, Development, and Evaluation (GRADE) (Guyatt et al., 2008). We assessed the risk of bias of included RCTs according to their research limitations, inconsistent results, evidence intervention or reporting bias. The reviewers independently described the evaluation of each field by evaluating each field and described it as “low risk,” “high risk,” or “unclear risk” of bias (Figure 3).

### Virologic suppression

For the key virologic suppression outcomes, we investigated the efficacy rate of LPV/r-based second-line therapy regimes when virologic suppression was <400 copies/mL after treatment. Nine studies [six (Cohen et al., 2005; SECOND-LINE Study Group et al., 2013; Paton et al., 2014; Ciaffi et al., 2015; La Rosa et al., 2016; Hakim et al., 2018) reporting randomized controlled trials and three (Fox et al., 2010; Hosseinipour et al., 2010; Patel et al., 2013) observational studies] for viral suppression were included in this meta-analysis. Seven (Cohen et al., 2005; Fox et al., 2010; Hosseinipour et al., 2010; Patel et al., 2013; SECOND-LINE Study Group et al., 2013; Ciaffi et al., 2015; La Rosa et al., 2016) trials reported viral suppression at 48 weeks, whereas two (Paton et al., 2014; Hakim et al., 2018) reported it at 96 and 144 weeks, respectively. The virologic suppression rate in these studies and the combined virologic suppression rate (48 weeks) are listed in Table 2. The virologic suppression rate of LPV/r-based second-line therapy was 77% (95% CI: 70–84%) at 48 weeks based on intention-to-treat (ITT) analysis in the 7 articles and was 87% (95% CI: 81–93%) according to pre-protocol (PP) analysis in the 4 articles at 48 weeks.

**Table 2:** Virologic response rates for second-line antiretroviral therapy patients using intention-to treat analysis and pre-protocol analysis (48 weeks).

	**Using ITT analysis**		**Using PP analysis**	
	**Range**	**Combined rate (95%CI)**	**Range**	**Combined rate (95%CI)**
LPV/r-based regimen	61.89–91.86%	77% (70–84%)	68.15–97.93%	87% (81–93%)
LPV/r+RAL regimen	82.59–91.96%	87% (78–96%)	92.53–97.93%	95% (90–100%)
LPV/r+NRTIs regimen	61.89–90.94%	74% (66–83%)	68.15–94.40%	81% (72–91%)

*NRTI, nucleoside/nucleotide reverse transcriptase inhibitor; RAL, raltegravir; LPV/r, ritonavir-boosted lopinavir*.

**Figure 4:**
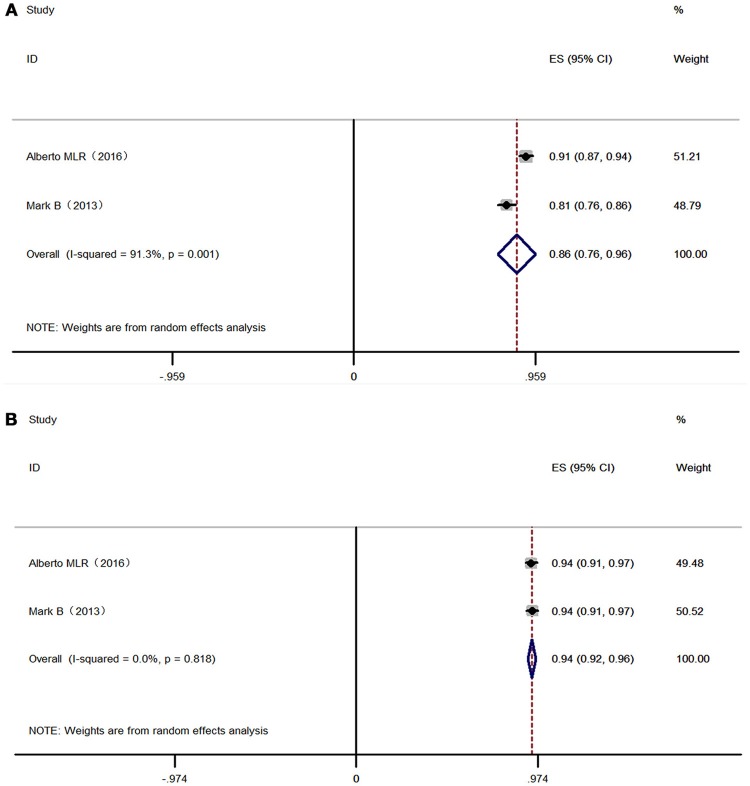
Forest plot of 2-3 NRTI plus LPV /r in virologic suppression. Each risk ratio represents the effect of the second-line treatment in people with HIV with previous ART failure. **(A)** Intention-to-treat analysis of HIV RNA <400 copies/mL. **(B)** Pre-protocal analysis of HIV RNA <400 copies/mL. NRTI, nucleoside/nucleotide reverse transcriptase inhibitor; LPV /r, Ritonavir-boosted lopinavir.

**Figure 5:**
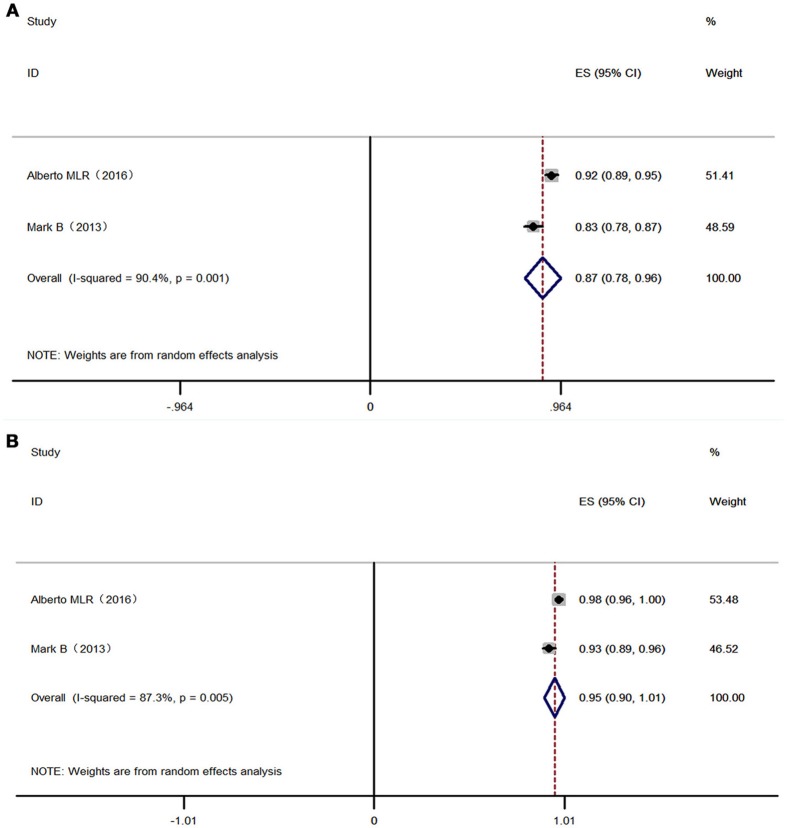
Forest plot RAL plus LPV /r in virologic suppression. Each risk ratio represents the effect of the second-line treatment in people with HIV with previous ART failure. **(A)** Intention-to-treat analysis of HIV RNA <400 copies/mL. **(B)** Pre-protocal analysis of HIV RNA <400 copies/mL. RAL, Raltegravir; LPV /r, Ritonavir-boosted lopinavir.

**Figure 6:**
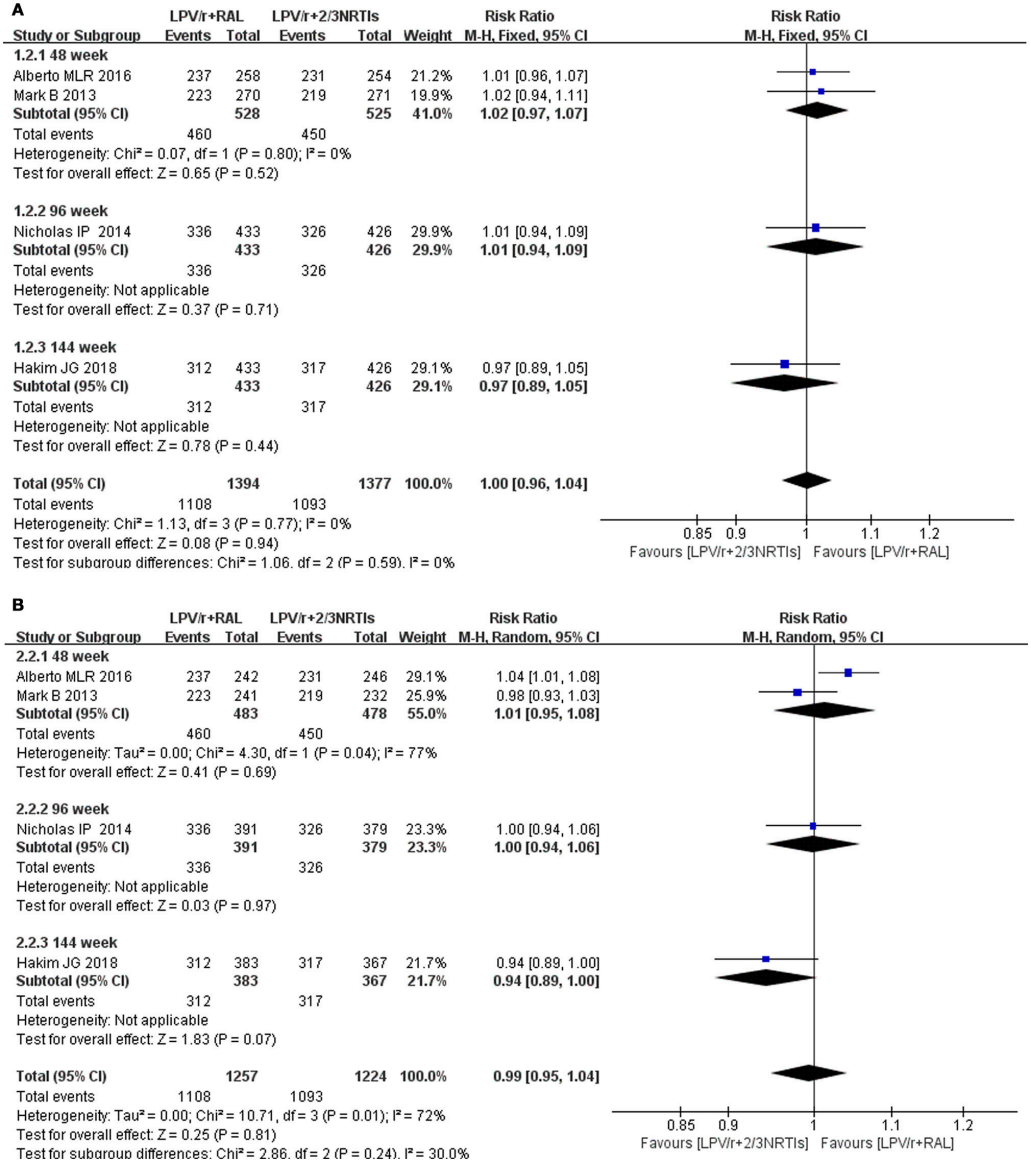
Forest plot of difference between 2-3 NRTI plus LPV/r and RAL plus LPV/r in virologic suppression. Each risk ratio represents the effect of the second-line treatment in people with HIV with previous ART failure. **(A)** Intention-to-treat analysis of HIV RNA <400 copies/mL **(B)** Pre-protocal analysis of HIV RNA <400 copies/mL. NRTI, nucleoside/nucleotide reverse transcriptase inhibitor; RAL, Raltegravir; LPV/r, Ritonavir-boosted lopinavir.

Overall, 525 patients in 2 studies using 2-3 NRTIs and LPV/r as second-line treatment showed a virologic suppression rate of 86% (95% CI: 76–96%) (ITT) and 94% (95% CI: 92–96%) (PP) in 48 weeks, while 528 patients using LPV/r plus RAL as a second-line treatment showed a virologic suppression rate of 87% (95% CI: 78–96%) (ITT) and 95% (95% CI: 90–100%) (PP) in 48 weeks (Figures 4, 5). There was no significant difference between LPV/r combined with RAL and LVP/r plus 2-3 NRTIs (the current standard of care second-line regimen) at 48 weeks, 96 weeks and 144 weeks (Figure 6).

### CD4 cell counts

**Figure 7:**
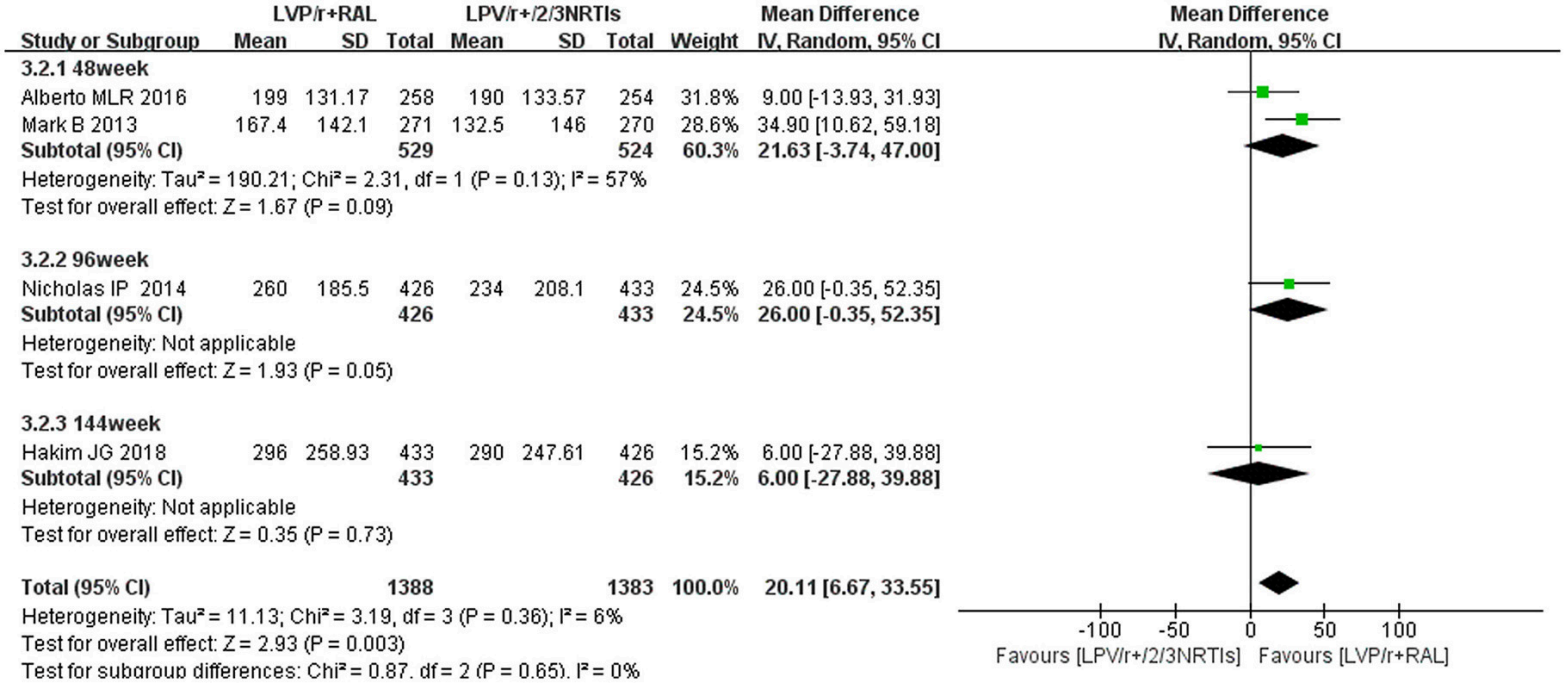
The difference between 2-3 NRTI plus LPV /r and RAL plus LPV /r in the change of CD4 cell counts. Data are the difference in mean CD4 cell count for LPV/r plus RAL vs. LPV/r plus NRTis. CI, credible interval; NRTI, nucleoside/nucleotide reverse transcriptase inhibitor; RAL, Raltegravir; LPV /r, Ritonavir-boosted lopinavir.

We analyzed changes in CD4 cell counts from baseline to 48 weeks, 96 weeks and 144 weeks in this study. In each study, the mean differences in CD4 cell count changes from baseline to different time points after treatment are shown in Table 1. Alberto MLR describes the CD4 cell counts as medians. We estimated the mean and standard deviation using median, extreme differences, and sample size (Hozo et al., 2005). Four of the nine studies referred to the CD4 cell counts after treatment with LPV/r plus RAL regimens. RAL combined with LPV/r did not result in a significantly higher increase in CD4 cell counts than 2-3 NRTIs plus LPV/r at 48 weeks (mean difference 21.63 cells per μL, 95% CI [−3.74, 47.00], *P* = 0.09), 96 weeks (mean difference 26.00 cells per μL, 95% CI [−0.35, 52.35], *P* = 0.05) and 144 weeks (mean difference 6.00 cells per μL, 95% CI [−27.88, 39.88], *P* = 0.73) (Figure 7).

### Safety and adherence of LPV/r-based second-line antiretroviral therapy

**Figure 8:**
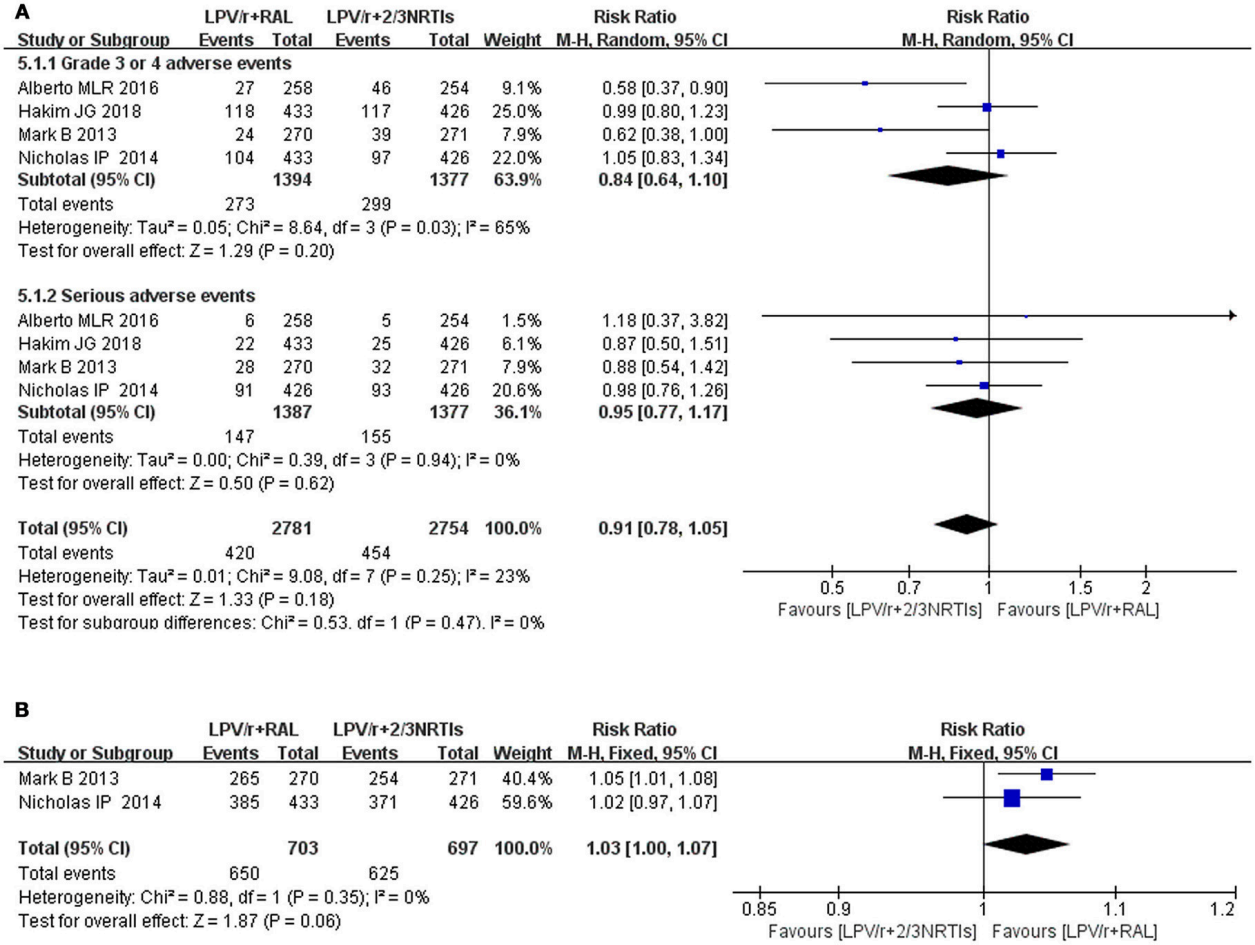
The different between NRTis plus LPV/r and RAL plus LPV/r in the change of grade 3 or 4 adverse events, serious events and adherence. Each risk ratio represents the effect of the second-line treatment in people with HIV with previous ART failure. **(A)** are desired for grade 3 or 4 adverse events and serious events, whereas **(B)** are desired for adherence. CI, credible interval; NRTI, nucleoside/nucleotide reverse transcriptase inhibitor; RAL, Raltegravir; LPV/r, Ritonavir-boosted lopinavir.

Seven studies mentioned the safety of LPV/r, and four (SECOND-LINE Study Group et al., 2013; Paton et al., 2014; La Rosa et al., 2016; Hakim et al., 2018) of the seven studies involved LPV/r plus RAL and LPV/r plus 2-3 NRTIs. In four randomized controlled trials assessing NRTI plus LPV/r and RAL plus LPV/r, we found no difference between treatment arms for grade 3 or 4 adverse events (RR 0.84, 95% CI 0.64, 1.10, *P* = 0.20) and serious adverse events (RR 0.95, 95% CI 0.77, 1.17, *P* = 0.62) (Figure 8A). In one randomized controlled trial of LPV/r and atazanavir (ATV) (Cohen et al., 2005), fasting LDL cholesterol levels were lower in the atazanavir group than in the LPV/r group at 48 weeks, although the former group had higher fasting LDL cholesterol levels than the latter group at baseline. In some studies, a decline in GFR was mentioned, and the reduction in eGFR between baseline and week 48 was >25% in 42 (14.1%) patients (Ciaffi et al., 2015). The eGFR of 33 (4%) patients was <60 mL/min/1.73 m^2^ compared with the RAL plus LPV/r group. The decrease in GFR in the NRTI plus LPV group was more obvious (Paton et al., 2014). Two studies (SECOND-LINE Study Group et al., 2013; Paton et al., 2014) involved adherence. We compared the adherence of LPV/r plus RAL and LPV/r plus NRTIs. Although the differences were not statistically significant (RR = 1.03, 95% CI [1.00, 1.07], *P* = 0.06), as seen from the figure, LPV/r plus RAL was superior to LPV/r plus NRTIs (Figure 8B).

### Drug resistance

The data on LPV/r resistance primarily came from clinical trials. Only 4 (SECOND-LINE Study Group et al., 2013; Paton et al., 2014; La Rosa et al., 2016; Hakim et al., 2018) of the 9 studies described the drug resistance mutations. Two studies (Paton et al., 2014; La Rosa et al., 2016) referred to the LPV/r resistance mutations in patients who failed with antiretroviral therapy. The Alberto MLR (La Rosa et al., 2016) study suggested that 96 participants (46 in the RAL plus LPV/r group, 50 in the NRTI plus LPV/r group) had virologic failure by the end of follow-up, and there were failure samples for genotype analysis for 80% of the participants. The major protease inhibitor mutations were 7% 46I, 4% 76V, and 4% 82A. The Hakim et al. (2018) study observed that 2% (7/321) of the participants (viral loads <1,000 copies/mL) showed PI-resistance mutations. The mutations were 46I and 54V (Table 3). For LPV/r, the most common protease inhibitor mutations were 46I, 54V, 82A/82F, and 76V. There was no participant that had virologic failure due to LPV/r resistance.

**Table 3:** Drug-resistant mutations in patients who failed antiretroviral therapy.

**Study name**	**NRTIs mutations % (n/N)**	**RAL mutations (n/N)**	**LPV/r mutations (n/N)**
Paton et al., 2014	Intermediate- or high-level resistance in 2% of patients in the NRTI group and 1% in the RAL group who failed antiretroviral therapy		
SECOND-LINE Study Group et al., 2013	14.0% (6/43)	14.9% (7/47)	Not given
La Rosa et al., 2016	13.3% (6/50)	26.09% (12/46)	15.56% (7/50)
Hakim et al., 2018	3% (10/321)	7% (10/321)	2% (7/321)

*N, the number of virologic failure patients; n, the number of patients with drug-resistant mutations. NRTI, nucleoside/nucleotide reverse transcriptase inhibitor; RAL, raltegravir; LPV/r, ritonavir-boosted lopinavir*.

## Discussion

With the widespread accessibility of ART for treatment of HIV-infected patients, poor adherence, drug toxicity and drug resistance has become a major concern associated with frequent clinical and virologic failure (Cohen et al., 2005). Two potential options for increasing viral suppression are ritonavir-boosted PIs and the incorporation of new and more potent agents into ART regimens (Cohen et al., 2005). After failure of a first-line NRTI-based regimen, a boosted PI plus 2 NRTIs is the strategy preferred by the WHO for second-line ART. As the first ritonavir-boosted PI, LPV/r has been widely used as a standard third drug in second-line regimens (World Health Organization, 2016).

Our analysis supported WHO guidelines (World Health Organization, 2016) for selection of second-line ART with a LPV/r-based regimen. In the ITT analysis for LPV/r-based second-line therapy, the viral suppression rate (HIV RNA <400 copies/mL) was as high as 77% at 48 weeks in the 9 articles. In the PP analysis, the viral suppression rate was 87% at 48 weeks. Our findings are based on many patients from RCT studies and observational studies, and thus, the results are more reliable than those of individual studies. The data showed that LPV/r-based second-line ART had good efficacy for patients who failed first-line ART.

In addition, four trials investigated the comparative efficacy between LPV/r plus RAL and LPV/r plus 2-3 NRTIs in second-line ART based on WHO guidelines. There were no significant differences between 2-3 NRTIs plus LPV/r and RAL plus LPV/r in virologic suppression and CD4 cell count recovery. Our meta-analysis indicated that RAL plus LPV/r was not inferior to standard second-line treatment (NRTI plus LPV/r) for patients with virologic failure. In patients who failed all NRTIs, LPV/r plus RAL is a good option. Further study is warranted to investigate whether similar outcomes can be achieved with LPV/r plus other integrase inhibitors as second-line ART regimens.

For two treatment regimens, there were no significant differences in grade 3 or 4 adverse events or serious events. In a study carried out by Cohen et al. (2005), grades 3 to 4 hyperbilirubinemia occurred in only less than 1% of patients treated with LPV/r. Compared with the NRTIs plus LPV/r groups, the RAL plus LPV/r regimen was well-tolerated, but total cholesterol and LDL were significantly increased. Thus, when using RAL plus LPV/r as a second-line ART regimen, more attention should be paid to dyslipidemia in patient.

The present review has multiple limitations. To reduce the possibility of missing key studies as much as possible, we searched three scientific databases and carefully reviewed the cited references of included studies, review articles and abstracts from recent conferences. However, it should be noted that the bulk of participants in our identified literature come from sub-Saharan African countries; few studies were contributed from other regions. Due to the lack of independence between study populations, our effectiveness assessment could be biased. There were two studies with the same participants and the same baseline characteristics, and the only difference was follow-up time. Some important outcomes, including CD4 cell counts and side effects, were limited by the very low number of events.

In conclusion, our meta-analysis showed that LPV/r as a second-line ART regimen had good efficacy and little drug resistance. RAL plus LPV/r as an alternative second-line regimen was not inferior to LPV/r plus NRTIs in viral suppression, immunological reconstitution, grade 3 or 4 adverse events, serious events or treatment adherence. However, it is worth noting that RAL plus the LPV/r regimen was more likely to cause dyslipidemia. Compared with RAL, LPV/r is available in resource-limited settings. In China, LPV is the first protease inhibitor prescribed. Compared with RAL, NRTIs and LPV/r are free of charge. The higher efficiency and lower side effects of LPV/r could achieve better therapeutic effect. The results of this systematic review clearly showed solid evidence that LPV/r-based ART is a preferred second-line therapy regimen and should be widely used in resource-limited countries. More clinical trials, particularly with an alternative second-line regimen of RAL plus LPV/r, are needed to better guide WHO treatment strategies and policy.

## Author contributions

YH and YL analyzed the data and wrote the manuscript. YZ, XT, and AS collected and prepared the samples. HC and YH performed the analyses. XH and YC designed the study and revised the manuscript. All authors critically revised the manuscript.

### Conflict of interest statement

The authors declare that the research was conducted in the absence of any commercial or financial relationships that could be construed as a potential conflict of interest.
